# Metallo-Beta-Lactamase-like Encoding Genes in Candidate Phyla Radiation: Widespread and Highly Divergent Proteins with Potential Multifunctionality

**DOI:** 10.3390/microorganisms11081933

**Published:** 2023-07-28

**Authors:** Mohamad Maatouk, Vicky Merhej, Pierre Pontarotti, Ahmad Ibrahim, Jean-Marc Rolain, Fadi Bittar

**Affiliations:** 1 Microbes, Evolution, Phylogénie et Infection (MEPHI), Institut de Recherche pour le Développement (IRD), Assistance Publique-Hôpitaux de Marseille (AP-HM), Aix-Marseille University, 13005 Marseille, France; mohamad.maatouk@etu.univ-amu.fr (M.M.); pierre.pontarotti@univ-amu.fr (P.P.); ahmad.ibrahim@etu.univ-amu.fr (A.I.); jean-marc.rolain@univ-amu.fr (J.-M.R.); 2 Institut Hospitalo-Universitaire (IHU) Méditerranée Infection, 13005 Marseille, France; 3 Centre National de la Recherche Scientifique (CNRS-SNC5039), 13009 Marseille, France

**Keywords:** Candidate Phyla Radiation, bacteria, metallo-beta-lactamase fold, massive analysis, functional diversity, sequence similarity network, protein domains, enzyme promiscuity

## Abstract

The Candidate Phyla Radiation (CPR) was found to harbor a vast repertoire of genes encoding for enzymes with potential antibiotic resistance activity. Among these, as many as 3349 genes were predicted in silico to contain a metallo-beta-lactamase-like (MBL-like) fold. These proteins were subject to an in silico functional characterization by comparing their protein profiles (presence/absence of conserved protein domains) to other MBLs, including 24 already expressed in vitro, along with those of the beta-lactamase database (BLDB) (*n* = 761). The sequence similarity network (SSN) was then used to predict the functional clusters of CPR MBL-like sequences. Our findings showed that CPR MBL-like sequences were longer and more diverse than bacterial MBL sequences, with a high content of functional domains. Most CPR MBL-like sequences did not show any SSN connectivity with expressed MBLs, indicating the presence of many potential, yet unidentified, functions in CPR. In conclusion, CPR was shown to have many protein functions and a large sequence variability of MBL-like folds, exceeding all known MBLs. Further experimental and evolutionary studies of this superfamily of hydrolyzing enzymes are necessary to illustrate their functional annotation, origin, and expansion for adaptation or specialization within a given niche or compared to a specific substrate.

## 1. Introduction

The Candidate Phyla Radiation (CPR) represents a significant portion of the microbial domain, encompassing more than 26% of known microbial diversity [1]. Genomic analyses have shown that these small microorganisms (100 to 300 nm) have a reduced genome size (around 1 Mbp) but great heterogeneity with regard to genome sequences [2,3,4]. They appear to be able to control their ecological niches by emitting and detecting communication molecules as a result of their rich repertoire of quorum-sensing proteins [5,6]. CPR members have an exosymbiotic or parasitic relationship with their bacterial host, which renders their culture in vitro extremely fastidious [5,7]. CPR members have been found in different environments around the world [8,9,10,11,12,13]. They have also been detected in the human microbiome through metagenomic analysis and 16S rRNA metabarcoding since they do not yet have a pure culturable representative [14]. CPR genomes contain a high percentage of genes that code for proteins with unknown functions; “orphan”, also known as hypothetical proteins [15]. A large comparative genomic study identified many functional domains that can confer antibiotic resistance activity in the hypothetical proteins [16]. New technologies, such as high-throughput methods, have largely contributed to the increasing number of new genomic sequences added to public databases, and powerful computational tools have created important links between molecular biology and enzymology [17]. However, despite many advances, the current data reveal significant gaps in knowledge on CPR, especially the functional role of hypothetical proteins and enzymes with antibiotic activity in terms of their colonization capacity and environmental expansion.

The rapid accumulation of sequences largely surpasses the analytical capabilities for function prediction to assign biological or biochemical roles. Only a tiny fraction of protein superfamilies has been experimentally characterized [18]. Thus, protein superfamilies with members that display close sequence identity and protein structure may contain various components with varyingly related functions [19]. Among the largest superfamilies that have been well studied is that of the beta-lactamases, which consists of thousands of known proteins, and a wealth of information on their properties is available [20,21,22]. Sequences with a beta-lactamase fold were initially found in bacteria resistant to beta-lactams and were named beta-lactamases. These enzymes are known for hydrolyzing the amide bond of the four-membered beta-lactam ring, which makes beta-lactam antibiotics inactive [23]. Beta-lactamases are divided into four groups: classes A, B, C, and D [24]. The class A, C, and D beta-lactamases are known as serine-beta-lactamases, and they use an active serine residue to attack the beta-lactam ring [24]. On the other hand, class B beta-lactamases, also called metallo-beta-lactamases (MBLs), utilize a zinc ion for their activity [25]. The MBLs are further divided into B1, B2, and B3, based on the number of zinc ions required for their activity. The B1 and B3 enzymes require two zinc ions to stabilize the hydroxide ion, which acts as a nucleophile to attack the beta-lactam ring. In contrast, B2 enzymes only require a single zinc ion for their activity [25].

Nevertheless, MBLs were revealed to be highly versatile proteins; they showed the ability to catalyze other reactions [26,27]. In a previous study by Baier and Tokuriki, the 24 studied enzymes of this superfamily with 15 distinct functional families were overexpressed, purified, and showed significant enzyme promiscuity [28]. These 24 enzymes were chosen based on their broad variety of sequence, structural, and functional diversity in the MBL superfamily. It is likely that these proteins switched from one function to another throughout evolution [27]. The majority of its members exhibit beta-lactamase activity, which suggests that it was present in the common ancestor of the beta-lactamase superfamily [29]. The acquisition of multiple functions within an enzyme superfamily is the cause of its expansion, as previously shown in various superfamilies, including isoprenoid synthase I, kinases, the crotonase family, and others [19,27,30,31,32]. Enzymes with an MBL fold were found in all life domains, including eucaryotes, human genomes, archaea, giant virus, bacteriophages, and, recently, in CPR [33,34,35,36,37]. They display great multifunctionality with a variety of specialized enzymatic and/or non-enzymatic activities depending on the ecological system within which they are established [33,38]. The literature has reported over thirteen activities of this fold, including nucleases, ribonucleases, glyoxalase, lactonases, phospholipases, and the degradation of ascorbic acid and anti-cancer drugs, as well as membrane transport [39]. Thus, the terminology used to identify proteins belonging to the same family may vary depending on the protein annotation method employed by researchers, which often relies on the initial sequence hits obtained from Blast analysis [18,40,41]. Therefore, it is conceivable that by employing more sensitive approaches in a functional manner, it is possible to search for distant homologous sequences by recognizing sequences that are too distant from existing ones, as previously indicated in studies regarding class A beta-lactamases [42,43]. Furthermore, protein similarity networks can provide summary views of unknown functional data in large protein superfamilies [28]. They are presented as a flexible in silico approach for identifying homologous MBL enzymes and predicting the functions of the uncharacterized proteins in unsuspected microorganisms [31].

A recent study showed that beta-lactamase-encoding genes were the most prevalent in the CPR resistome [16]. These were mainly annotated as hypothetical proteins [7,44]. Five of these proteins revealed a hydrolase activity against several beta-lactam substrates (penicillin G, amoxicillin, and ampicillin) when using liquid chromatography–mass spectrometry. Moreover, they showed RNase activity in both the presence and absence of the beta-lactamase inhibitors EDTA chelator and sulbactam [44]. Hypothetical proteins are often disregarded due to their structural and low sequence similarities with well-characterized proteins. Similar mis-annotations in enzyme superfamilies are commonly associated with the overprediction of a molecular function, annotated using automated computational analysis, following high-throughput genome sequencing [18]. Thus, error percolation may occur in the functional annotation of these new protein sequences due to insufficient investigation [45,46]. Therefore, the challenges of in silico approaches in addressing and predicting the functions of uncharacterized proteins in a superfamily, such as beta-lactamase, may be particularly a fruitful area of research. Beta-lactamases have been proven to be one of the best pools of proteins for identifying and deciphering new functions [15]. The role of the recently discovered beta-lactamase-like sequences in CPR genomes does not appear to be fully understood, but deserves to be explored, particularly as CPR genomes have been detected in old samples as with beta-lactamases [12,37].

The main aim of this study was to gain access to address the functional relationships between sequences carrying the metallo-beta-lactamase-like (MBL-like) fold detected in CPR cells in comparison to other known MBLs, including those expressed in vitro. This comprehensive analysis of protein sequences on a large scale is useful to explore the functional diversity of MBL-like sequences in CPR genomes and to try to understand their role in evolution.

## 2. Results

### 2.1. CPR MBL-like Sequences: Different Sizes and Divergent Sequences

**Figure 1:**
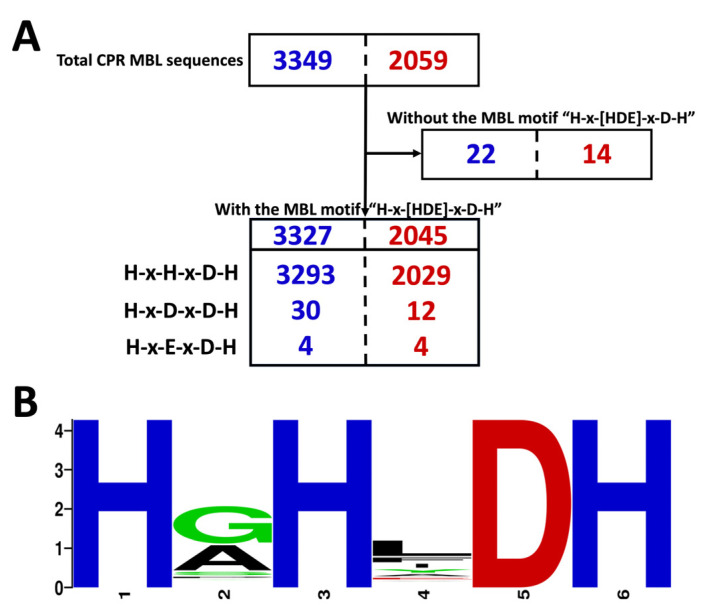
CPR sequences with a metallo-beta-lactamase-like (MBL-like) fold. (**A**) Overview of the total number of CPR sequences with an MBL-like fold in blue (with duplication) and in red (without duplication/unique sequences). (**B**) Signature logos showing the different motifs of the CPR MBL-like fold. The data for these logos consist of 3327 CPR sequences with the group-specific motifs “H-x-[HDE]-x-D-H”, where “x” can be any amino acid. The logos were generated using the WebLogo online tool version 2.8.2.

The 3349 CPR MBL-like sequences identified in 2427 genomes were described in detail. Among these, the MBL-like sequences in the B3 subclass represent 88% (*n* = 2954) and they were found in all CPR superphyla, unlike those of B1 and B2 (see the Section 4). We found that CPR genomes can harbor many sequences with the MBL-like fold (up to seven in the superphylum Parcubacteria), and 67% (*n* = 1634) and 28% (*n* = 691) of the genomes showed one and two MBL-like sequences, respectively (see Table S1 in the Supplemental Material). After manual screening to remove redundancy/duplication of all the CPR MBL-like sequences, 2059 unique sequences with an MBL-like fold were found in different CPR genomes (Figure 1). More than 75% (*n* = 1554) of the unique sequences appeared only once in our results. The remainder were found in many genomes (see Table S1 in the Supplemental Material). A single sequence of 624 amino acids was found up to 20 times in 20 genomes in the only superphylum of *Candidatus* Parcubacteria (Table S1). This was also the case for all other duplicated sequences, where redundancy occurred exclusively among CPR genomes of the same superphylum. Hence, no duplications of a protein sequence with an MBL-like fold were found between the different tested CPR superphyla.

Among the unique CPR MBL-like sequences (*n* = 2059), 99% (*n* = 2045) had the motif “H-x-[HDE]-x-D-H” (Figure 1). We found that the majority of the MBL-like sequences (*n* = 2029) had the motif “H-x-H-x-D-H” where the first “x” was “G” in 47% of the data (*n* = 1548) and “A” in 32% of the sequences (*n* = 1062) (Figure 1). The second variable “x” was “L” in 35% of the sequences (*n* = 1154) (Figure 1). Therefore, the most common motif was “H-G-H-L-D-H”, which was found in 21% of sequences (*n* = 691), followed by “H-A-H-L-D-H” and “H-G-H-F-D-H” in 11% (*n* = 346) and 10% (*n* = 329), respectively (Figure 1). The remaining 16 CPR sequences (34 copies) had the motif “H-x-[DE]-x-D-H” (Figure 1). However, there was no actual correlation between the character of the MBL-like motif identified in the protein sequences and the superphylum of CPR to which they belong. Interestingly, 14 MBL-like sequences (22 copies) did not have the group-B-specific pattern “H-x-[HDE]-x-D-H” (Figure 1). It is noteworthy that the distribution of unique and duplicated CPR MBL-like sequences across different motifs was the same. Therefore, the multiplication of these sequences in the CPR genomes did not significantly highlight a specific repeated type of sequence or a specific character in the MBL-like motif between CPR genomes.

**Figure 2:**
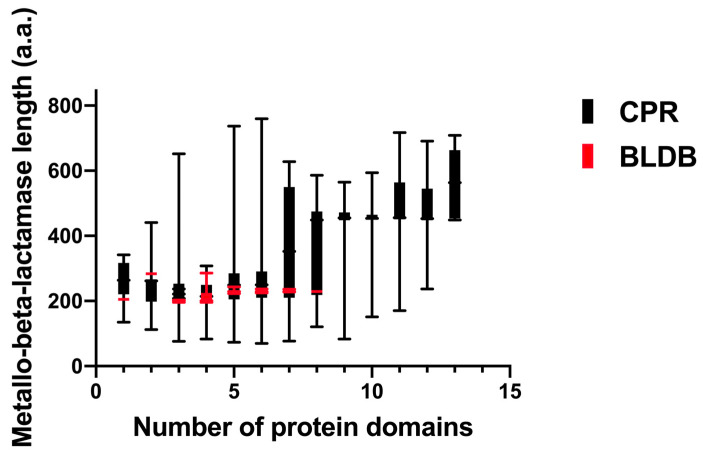
Boxplot showing the length of metallo-beta-lactamase (MBL) sequences of the beta-lactamase database (BLDB) and CPR MBL-like sequences, based on the number of protein domains detected according to the Conserved Domain Database (CDD) search. CPR sequences are shown in black while those of BLDB are in red.

**Figure 3:**
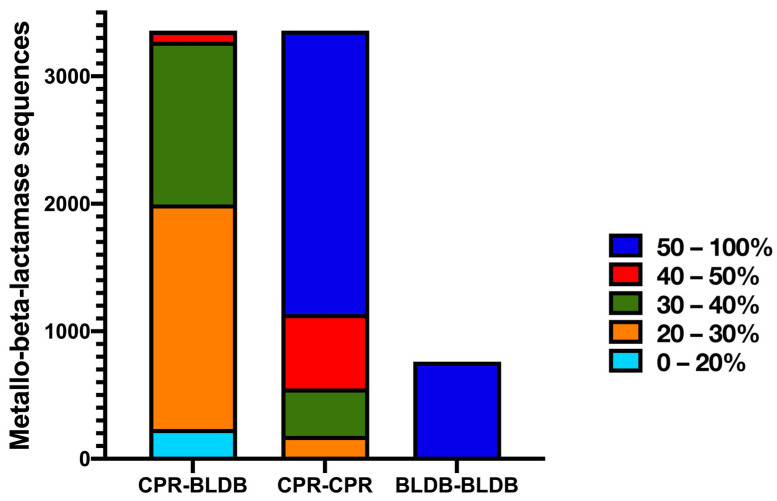
Histogram showing the identity percentage between CPR metallo-beta-lactamase-like (MBL-like) sequences, MBL sequences of the beta-lactamase database (BLDB), and between these two groups of sequences.

Furthermore, the length of the identified unique CPR MBL-like sequences had an average of 425 amino acids, ranging from 70 to 1038 amino acids (Figure 2). These sequences were almost two-and-a-half times longer than the bacterial sequences of the beta-lactamase database (BLDB) (average length of 266 amino acids [ranging from 235 to 428 amino acids]) (Figure 2). MBL-like sequences from CPR showed a small percentage of similarity, ranging from 20% to 40%, with those from bacteria (Figure 3). This high divergency in protein sequences was also found among CPR MBL-like sequences in all superphyla (with a mean of 44.9% [18.75% to 100%] of similarity percentage) (Figure 3). In contrast, there was a high similarity percentage between MBL sequences for those of BLDB (with a mean of 94.6% [34.7% to 99.6%]) (Figure 3).

### 2.2. The CPR MBL-like Sequences Are Rich in Various Protein Functions

**Figure 4:**
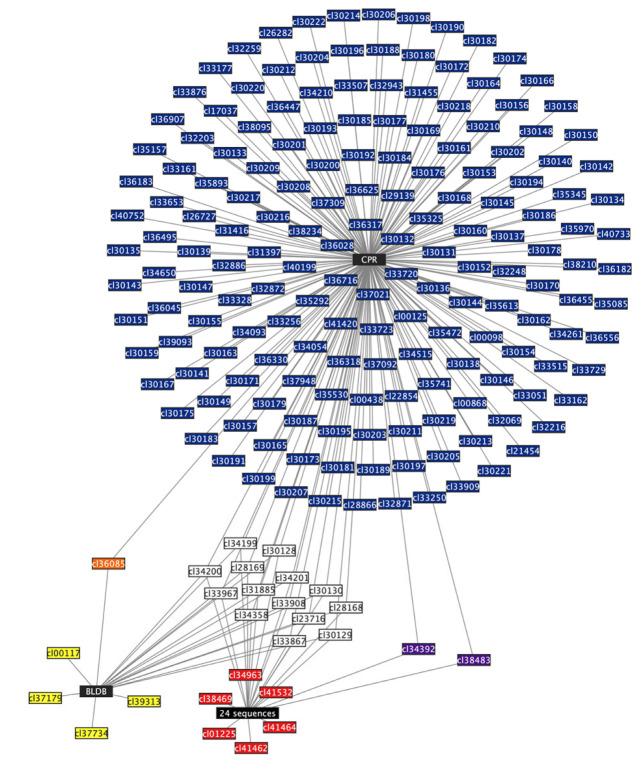
Network showing the protein domains found in the “cl23716” fold of metallo-beta-lactamase (MBL) sequences from the three analyzed groups. Each node (rectangle) represents a protein domain. Black nodes represent the name of the group sequences with an MBL fold. Dark blue nodes represent a protein domain found only in CPR MBL-like fold sequences. Red nodes represent a protein domain found only in the MBL fold of the 24 expressed sequences in vitro. Yellow nodes represent a protein domain found only in the MBL fold of beta-lactamase database (BLDB) sequences. White nodes represent protein domains found in the three analyzed groups of MBL fold sequences. Purple nodes represent a protein domain found in the CPR MBL-like fold and the 24 expressed sequences. Orange nodes represent a protein domain found in CPR MBL-like fold sequences and those from BLDB. Each edge (line) between two nodes indicates the presence of the protein domain to the group to which the MBL fold is connected. This network does not represent a quantitative correlation.

Most CPR MBL-like sequences were annotated as sequences with an MBL fold and ribonuclease activity (*n* = 2601; 77.7%), while 18.5% of the sequences (*n* = 619) were annotated as hypothetical proteins, and 3.8% (*n* = 129) as other functions (Table S1). This protein annotation was performed through the Rapid Annotation using Subsystem Technology (RAST) server. Moreover, when using Phyre2, all 3349 MBL-like proteins found in CPR genomes showed homology and/or analogy, with high confidence, to other proteins with beta-lactamase functions. These functions include proteins with hydrolase activity, ribonuclease, oxidoreductase, carboxypeptidase, penicillin-binding protein, metallo-beta-lactamase like protein, and other functions (see Table S2 in the Supplemental Material). These CPR MBL-like sequences, with the 24 MBLs expressed in vitro (*n* = 24) and the MBL sequences from BLDB (*n* = 761), were found to exhibit a conserved and common functional protein fold according to the Conserved Domain Database (CDD) search (see Table S3 in the Supplemental Material). This common fold, “cl23716”, has a metallohydrolase activity that carries out multiple biological functions. For this purpose, we again searched for the presence of different protein domains in this common fold, “cl23716”, by reusing the CDD search (Table S3). Our findings showed that the CPR MBL-like sequences had the highest number of different types of protein domains (*n* = 101, mean = 8 domains/sequence [1 to 31]) compared to the expressed one (*n* = 22, mean = 2.5 domains/sequence [2 to 7]) and those of BLDB (*n* = 19, mean = 3.6 domains/sequence [1 to 8]) (Figure S1; Table S4). Moreover, fourteen domains were found in all three analyzed groups; they were responsible for L-ascorbate metabolism of protein UlaG, hydroxyacylglutathione hydrolase (glyoxylase II), and ribonuclease activity (Figure 4 and Figure S1; see also Tables S3 and S4). Aside from these fourteen domains, only two protein domains (for hydroxyacylglutathione hydrolase and alkyl sulfatase activity) were common between CPR and the expressed sequences, and one domain (for nitric oxide reductase activity) was common between the CPR and BLDB sequences (Figure 4 and Figure S1; see also Table S3). Finally, there was no common domain between BLDB and the expressed sequences that were not found in the CPR MBL-like sequences.

However, each group in the MBL superfamily analyzed in this study had unique protein domains that did not appear in the other groups. Starting with the BLDB group, its sequences included four domains for PDZ domains by which they were involved in the scaffolding of supramolecular complexes (Figure 4 and Figure S1; Table S3). For the expressed sequences, they had exclusively six domains involved in binding sterols, glucan, and choline, and a domain with pneumococcal surface protein A and alkyl sulfatase activity (Figure 4 and Figure S1). Thereafter, the CPR MBL-like group had as many as 84 protein domains that were not detected in the two other analyzed groups (Figure 4 and Figure S1). These 84 protein domains have a variety of functions, including reductase function for NADPH, thioredoxin, and selenate, as well as phospholipase, phosphatase, and other functions (Figure 4; see also Table S3 in the Supplemental Material). It is notable that different families within the MBL superfamily can share the same function and activity, even though their sequences have very low similarity when compared to one another. This was shown repeatedly in our results. For example, this was the case for the two domains “cl34392” and “cl35325”, which had two different terminologies in the CDD search but were coded for “alkyl sulfatase” activity, and which have sequences with a slight similarity of 14%. This was also the case for “cl36085” and “cl35325”, which shared the same function, “nitric oxide reductase”, with 36% similarity between sequences.

### 2.3. CPR MBL-like Sequences Have Different Protein Profiles and Many Potential Functions

**Figure 5:**
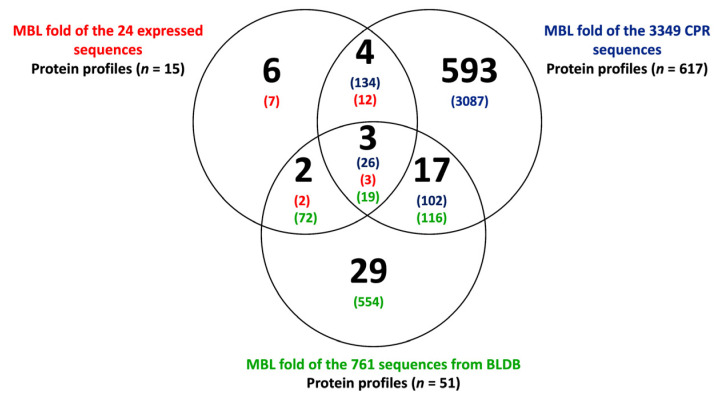
Venn diagram showing the number of protein profiles found in the three analyzed groups of metallo-beta-lactamase (MBL) fold sequences. The numbers in parentheses represent the number of MBL fold sequences found in each analyzed group.

Using the CDD search, we were able to assign 111 different protein domains to the 4134 MBL sequences of the three tested groups. When comparing the different MBL sequences, we were able to generate 654 signature profiles (Figure 5; see also Table S4 in the Supplemental Material). Significantly, the 3349 MBL-like sequences from CPR had the highest numbers, with a total of 617 different functional profiles, while those of the 761 BLDB had 51 profiles, and the 24 expressed sequences had only 15 different protein profiles (Figure 5). Only three protein profiles were common between the three groups: CPR, the expressed sequences, and BLDB, with 26, 3, and 19 sequences, respectively (Figure 5). The protein domains were differentially distributed among the remaining MBL sequences. Thus, 134 and 102 CPR sequences shared 4 and 17 protein profiles with 12 sequences of those already expressed in vitro and 116 BLDB sequences, respectively (Figure 5). There were only 2 common protein profiles between 2 expressed sequences and 72 of BLDB (Figure 5). It is notable that CPR had a significant number of unique protein profiles (*n* = 593) in comparison to 29 unique profiles in BLDB (*n* = 554 sequences) and 6 in the expressed sequences (seven sequences) (Figure 5). These unique CPR protein profiles were found in 92% of MBL-like sequences (*n* = 3087) (Figure 5).

**Figure 6:**
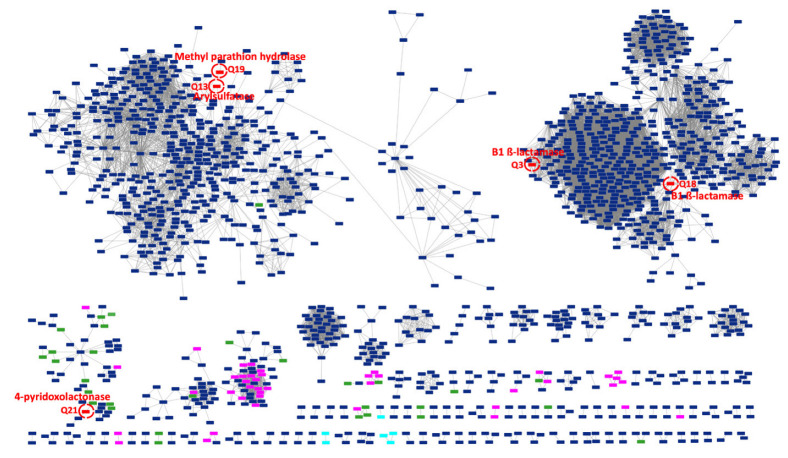
Sequence similarity network (SSN) of no redundant CPR metallo-beta-lactamase-like (MBL-like) fold “cl23716” sequences (*n* = 1932) with those of the 24 expressed sequences. Each node (rectangle) represents a group of 1–20 MBL-like fold sequences. The total nodes resulting from the SSN generated represents a total of 3373 MBL-like fold sequences. A red node surrounded by a red circle represents an expressed MBL sequence (*n* = 5 expressed sequences (Q3, Q13, Q18, Q19, Q21)). A pink node represents a CPR MBL-like fold that shares a protein profile with the expressed sequences. A green node represents a CPR MBL-like fold that shares protein profiles with those from the beta-lactamase database (BLDB). A light blue node represents a CPR MBL-like fold that shares a protein profile with the other two analyzed groups. A dark blue node represents a CPR MBL-like fold with a unique protein profile. Nodes with no connectivity (*n* = 436 representing 555 CPR MBL-like fold sequences) are not represented in this SSN. Each edge (line) between two nodes indicates that the sequences represented by the connected nodes have a BLASTp similarity percentage of 60% or more.

We mapped the large dataset of MBL-like sequences from CPR onto clusters of those expressed in vitro in the sequence similarity network (SSN) to visualize their connectivity. By integrating CPR sequences into the expressed sequences, we were able to distinguish those which were not yet characterized, allowing us to make functional hypotheses. By applying the appropriate parameters for the SSN, 426 MBL-like sequences from CPR (a total of 545 duplicated sequences) showed no connectivity with any of the analyzed queries. Only 35 CPR sequences showed connectivity with 5 sequences of the 24 expressed in vitro, demonstrating the small inter-relationships between the analyzed MBL-like sequences and the functional verification in vitro (Figure 6). The protein Q18 had the most edges (*n* = 23) with CPR sequences which were predicted to encode for ribonuclease. The protein Q21 had 4-pyridoxolactonase activity and was connected to seven CPR sequences annotated as beta-lactamase proteins (Figure 6). The remaining MBL-like sequences from CPR were annotated as hypothetical proteins which were connected to a Q13 sequence with arylsulfatase activity (*n* = 2), Methyl parathion hydrolase (Q19 with *n* = 2), and one to Q3 for the subclass-B1 MBL superfamily (Figure 6). Finally, another 2769 MBL-like sequences from the CPR were very dissimilar to the queries from the MBL expressed in vitro, yet they showed strong connections among themselves, reaching a maximum of 268 edges (Figure 6). The SSN, therefore, helped to group the CPR MBL-like sequences into more than 95 clusters but showed as many as 436 nodes without any connectivity (Figure 6).

These results corroborate the high diversity of MBL sequences and the multiplicity of their functional domains in the literature. The determination of protein profile signatures thus complements the SSN approach. CPR sequences with no connectivity but which share protein profiles with BLDB sequences could reflect a beta-lactam resistance function. Moreover, the connection between these CPR sequences and other CPR MBL-like sequences may suggest beta-lactam hydrolase activity (Figure 6).

## 3. Discussion

Superfamily analysis has been shown to provide a convenient context for addressing the daunting challenge of assigning function to uncharacterized proteins [17]. MBL is a very large superfamily that includes sequences that share the common fold of “MBL”, yet they contain many active site residues that can be involved in various functions with distinct mechanisms against various substrates [47]. Like other superfamilies, the MBL superfamily shows great functional diversity with several protein constituents that share very few common features [48]. It has been reported that the sequence similarity percentage among MBL sequences could be as low as 5% [49,50,51]; in particular, the sequences of subclass B3, which may have only nine conserved residues [25]. This explains the huge diversity of CPR MBL-like sequences, most of them belonging to subclass B3. Interestingly, low sequence similarity does not always mean different functional properties, since these genes may have a similar tertiary structure [52,53]. This is the case among the same family members which share similar three-dimensional structures and functions and use the same mechanism to catalyze the same overall reaction [48]. This was verified by using the search for specific motifs and 3D-structure prediction to confirm the MBL membership of the sequences analyzed here. CPR MBL-like sequences without the typical “H-x-[HDE]-x-D-H” motif may have a specific CPR motif which has not been identified at this stage. Crystallographic studies are needed for full recognition.

Many MBL proteins were found to contain a number of domains that help to enlarge the panel of activity of these proteins in comparison to their homologs, which have a single domain [54]. Thus, MBL sequences seem to be a common scaffold that has been exploited continuously throughout evolutionary history in natural environments to catalyze and/or hydrolyze a wide variety of chemical reactions [25,37,50,55,56]. This functional diversity is achieved structurally by adjusting the loops and patterns at the active site to suit different substrates. The various additional functional domains allow for a wide diversification of substrates, products, and cofactors, as well as great metabolic adaptability to various ecological niches [25]. This is critical for microorganisms present throughout the environment and detected in different clinical samples, such as CPR members [56]. Our results have shown a high number of protein domains within the MBL-like fold “cl23716” sequences from CPR in accordance with their widespread presence in the environment. It is noteworthy that the CPR MBL-like sequences were relatively long, reaching twice the size of the non-CPR sequences. It has been demonstrated that the multidomain expansion was consistent with the large protein size feature [54]. Finally, by searching for functional domains and organizing them into profiles, we were able to distinguish many different groups of functions for the tested sequences, including the fifteen enzymatic activities given by Baier and Tokuriki for their 24 studied MBL sequences [28]. This strong correlation with an in vitro method highlights the high predictive quality of our in silico method. Thus, MBL-like proteins from CPR have revealed a very diverse functional potential for degrading/hydrolyzing many compounds, going beyond their role in beta-lactam resistance [44]. The MBL superfamily may be an essential feature of the reduced genomes of the widespread CPR and should be tested in vitro.

The SSN-generated network allowed us to visualize all relationships within the large set of MBL-like sequences by grouping them into functionally related sequences in an intuitively accessible manner [31]. This combination of the signature-profiling of protein domains and the SSN method allowed us to predict the functions of some CPR sequences that shared the same protein profile and/or had a sequence similarity connection with the expressed MBL. Nevertheless, the results from SSN showed no significant connectivity between CPR MBL-like sequences and the 24 MBL expressed sequences, although a large number of clusters were identified in the SSN generated here compared to the network generated for the bacterial MBL superfamily [28]. Thus, from the CPR sequences with no connectivity in our SSN, we could predict the functions of 164 MBL-like sequences because of their sharing seven protein profiles with the expressed sequences. This functional prediction of CPR sequences is limited by our in silico analysis. The reported activity has not been validated yet by in vitro tests; experimental works are necessary to effectively characterize the function of these proteins. However, as SSN cannot be applied to infer evolutionary history, these findings indicate a high divergence among sequences, probably in relation to a large phylogenetic distance [31]. Much like taxonomic studies based on genome analysis, CPR MBL-like sequences seem to be very particular and allow us to classify CPR into a distinctive group, independent of other life domains [57,58]. Recent studies reported that distinct environments and microorganisms are reservoirs for novel beta-lactamase encoding gene precursors [16,44,59,60,61,62,63]. This large dataset of uncharacterized MBL-like sequences from CPR could present an ideal candidate to elucidate this functional diversity. Functional exploration can focus on the in vitro test of one sequence from each cluster to enable a functional prediction of all sequences from this cluster. Given that MBL proteins are promiscuous enzymes, there is a strong chance of finding their native activity when studying the MBL-like sequences from CPR genomes.

Our findings contribute to the comprehension of the potential multifunctionality exhibited by the MBL-like sequences found in CPR genomes, extending beyond their involvement in beta-lactam resistance. This study suggests that these enzymes play a pivotal role in essential biological processes within CPR cells, contributing to the survival strategies and microbial adaptation in different ecosystems. The presence of the MBL-like fold in CPR could be associated with the communication mechanisms employed by these microorganisms during obligatory symbiotic interactions with bacteria [57]. Alternatively, this fold may facilitate the degradation of a diverse range of substrates, allowing for the subsequent utilization of the resulting metabolites as valuable nutrients or carbon sources, like certain bacterial species [64,65]. However, the wide distribution of the MBL-like fold highlights the imperative for future in vitro investigations aimed at unraveling their origins and the extent of their functional diversity.

## 4. Materials and Methods

### 4.1. CPR MBL-like Sequences

As material for this study, we used our recently described CPR resistome [16], and selected all protein sequences of the beta-lactamase class B superfamily that contain the MBL-like fold, as identified using the National Center for Biotechnology Information (NCBI) CDD search. In this previous study, all CPR beta-lactamase-like encoding genes (*n* = 5759) included CPR serine-beta-lactamase-like sequences of classes A, C, and D and beta-lactamase-like sequences of class B [16]. The MBL-like sequences represent 58.32% (*n* = 3349) of all CPR beta-lactamases and they were divided into B1 (17 proteins), B2 (378 proteins), and B3 (2954 proteins) sub-classes [16]. The CPR genomes that encode for enzymes with the MBL-like fold were assigned according to NCBI taxonomy into thirteen CPR superphyla (*Candidatus* Parcubacteria (*n* = 1501), *Candidatus* Microgenomates (*n* = 509), *Candidatus* Saccharibacteria (*n* = 79), unclassified Patescibacteria group (*n* = 101), Candidate division WWE3 (Katanobacteria) (*n* = 69), *Candidatus* Peregrinibacteria (*n* = 57), *Candidatus* Berkelbacteria (*n* = 30), *Candidatus* Dojkabacteria (*n* = 32), *Candidatus* Doudnabacteria (*n* = 22), *Candidatus* Gracilibacteria (*n* = 4), *Candidatus* Absconditabacteria (*n* = 4), Candidate division Kazan-3B-28 (*n* = 10), and *Candidatus* Wirthbacteria (*n* = 2)) [16].

A manual search for the functional motifs was performed for each CPR sequence in order to identify the beta-lactamase-like sequences in the class B sub-groups (B1, B2, and B3). We used the pattern (H-x-[HDE]-x-D-H, where x can be any amino acid) as a group-specific motif to detect sequences of the MBL superfamily with high sensitivity (> 82%) and high specificity (100%) [66].

Further in silico analysis based on the 3D structure of CPR MBL-like sequences was conducted to achieve better stringency in identifying its beta-lactamase activity. Therefore, each sequence was screened through the Phyre2 online tool [67,68].

### 4.2. Data Set Curation for Protein Function Profiling

In order to compare the CPR MBL-like sequences with other MBL sequences, we retrieved the 24 expressed MBL sequences in vitro from the Swiss-Prot database (manually annotated and reviewed) [28] and the MBL sequences from the beta-lactamase database (BLDB) (all MBL available up to 1 November 2021) (*n* = 761) [69]. We identified the different functional domains that are present in the common “cl23716” MBL fold of all query sequences (*n* = 4134) using the CDD search with a maximum e-value of 0.0001. In this paper, we always refer to similar functional domains (with the same accession number), when conserved patterns of amino acids were detected in a protein query sequence based on local multiple sequence alignments of NCBI CDD search. The analysis of the different protein sequences of the MBL superfamily for the three groups of sequences enabled the identification of the common and unique protein domains found in each sequence. We were thus able to identify distinct protein profiles after manual comparison of the protein domains found in the different MBL sequences examined in this study. When these sequences contained both the same number and the same protein domains, we grouped all sequences into a single protein profile or family.

### 4.3. Construction of the Sequence Similarity Network

To fully understand the functional diversity of the MBL-like sequences found in CPR genomes, we evaluated its structure–function relationships with MBL sequences already expressed in vitro. For this purpose, we generated a sequence similarity network (SSN) for CPR sequences with the 24 MBL sequences expressed in vitro [28]. This was performed by an in-house all versus all protein BLAST of 3349 CPR sequences plus the 24 expressed sequences with a maximum e-value of 0.0001. All sequences used in the SSN were those of the common “cl23716” fold detected by the CDD search and cut from the initial sequences. They were then subjected to a sequence similarity search for possible homologs. The SSN based on alignments covering the length of the domain in common with a sequence identity greater than 30% were retained for further exploration [31]. Here, edges corresponding to pairwise relationship hits were filtered based on an expectation value that included a minimum similarity threshold of 60%. The clustering patterns of the SSN in this work were visualized using the Organic layout in Cytoscape version [3.9.1.].

## 5. Conclusions

In conclusion, this study sheds light on the functional diversity exhibited by MBL-like encoding genes in CPR genomes. Our results indicate that these sequences are longer and more diverse than bacterial MBL sequences, possessing a high content of functional domains. Thus, the SSN showed the presence of many potentials (high number of clusters), yet unidentified functions (no connectivity with expressed MBLs). They have potential multifunctionality unrelated to antibiotic resistance, which may play a major role in how CPR can adapt to their various environments. We suggest not limiting the MBL function to the hydrolysis of beta-lactam but instead referring to it as “metallo-hydrolases”. This would avoid ambiguity in the annotation of these enzymes and their automatic assignment to only the resistance function towards beta-lactam molecules.

Our thorough exploration serves as a starting point for future research projects for investigating the functions and the utility of MBL-like folds in CPR cells. Similar extensive analyses are expected to reveal even greater levels of enzyme promiscuity and functional connectivity in the MBL superfamily. This would increase our understanding of the metabolism of CPR members and would help to improve future culture assays for these fastidious and unusual microorganisms [70]. Finally, when considering the low patchiness of MBL among microbes and their presence in high phylogenetic depth, including in old metagenomes, MBL in CPR members are likely to be ancient and conserved with few loss events [12,37]. Further evolutionary and experimental studies are needed to elucidate the origins of MBL, its expansion, and diversification in the emergence of the CPR.

## Data Availability

Not applicable.

## Supplementary Materials

The following supporting information can be downloaded at: https://www.mdpi.com/article/10.3390/microorganisms11081933/s1. Figure S1: Venn diagram representing the number of different types of protein domains found in the metallo-beta-lactamase-like fold from the three analyzed groups of sequences; Table S1: Table showing data of the metallo-beta-lactamase-like fold sequences found in CPR genomes; Table S2: Table showing the results of the 3D structure analysis of CPR metallo-beta-lactamase-like sequences using the Phyre2 online tool; Table S3: Table showing the different types of protein domains found in the metallo-beta-lactamase-like fold “cl23716” from the three analyzed group of sequences; Table S4: Metadata table showing the different protein profiles of all metallo-beta-lactamase-like fold sequences from the three analyzed groups.
