# Supplementary figures and images for: Metallo-Beta-Lactamase-like Encoding Genes in Candidate Phyla Radiation: Widespread and Highly Divergent Proteins with Potential Multifunctionality
Source: Microorganisms. 2023 Jul 28;11(8):1933. doi: 10.3390/microorganisms11081933 (PMC10459063; doi:10.3390/microorganisms11081933)

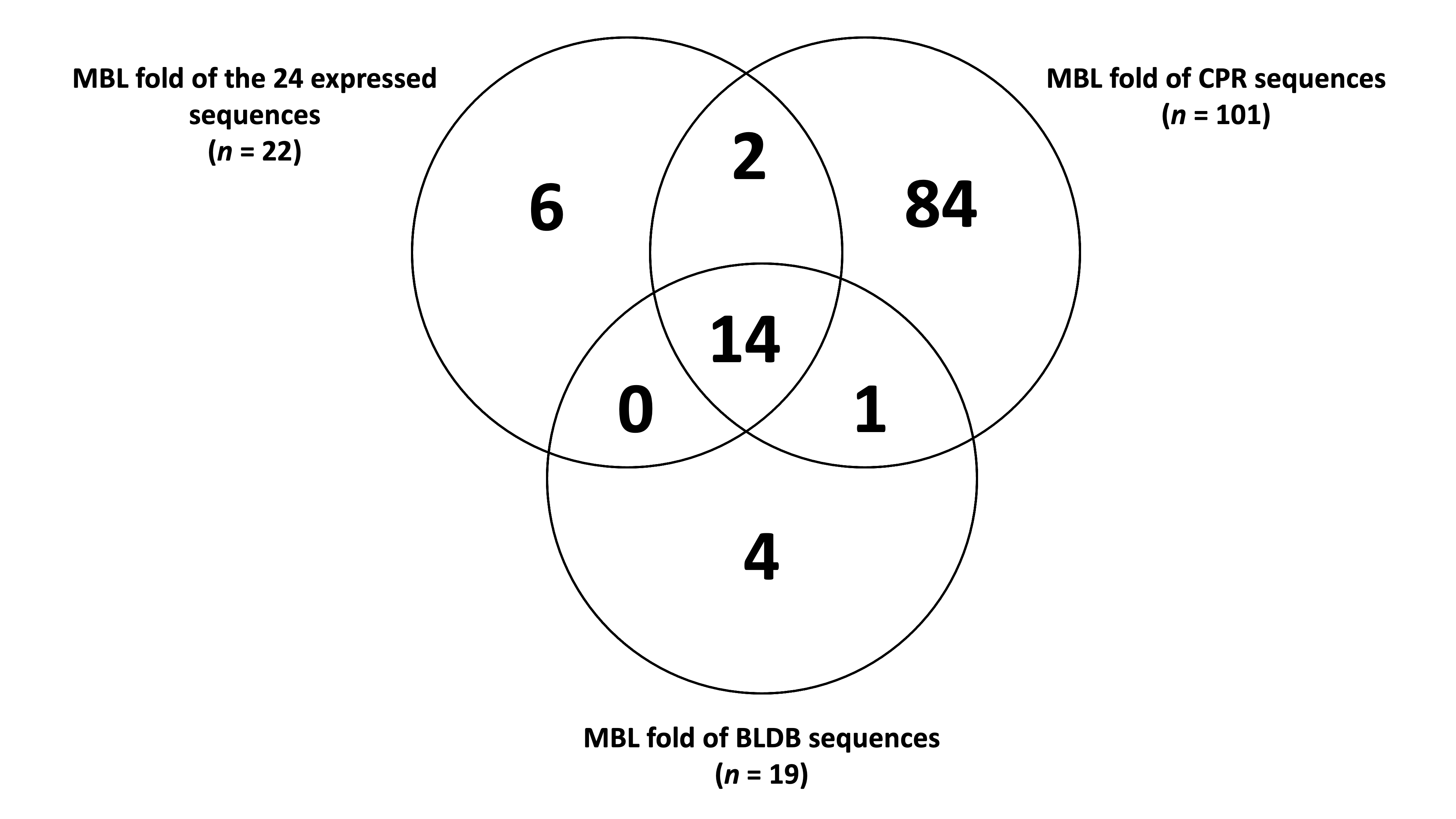

Supplement: Supplementary file 1 [file microorganisms-11-01933-s001.zip › Figure S1.tiff]
